# Saccular common iliac artery aneurysm associated with HLA-A26 and HLA-B27 in a young adult patient: a surgical case report and literature review

**DOI:** 10.1186/s44215-026-00250-9

**Published:** 2026-04-01

**Authors:** Hiroto Yasumura, Kenichi Arata, Koichiro Shimoishi, Yuki Ogata, Tomoyuki Matsuba, Yoshihiro Fukumoto, Goichi Yotsumoto, Yoshiharu Soga

**Affiliations:** 1 Department of Cardiovascular Surgery, Kagoshima City Hospital, 37-1 Uearatacho, 890-8760 Kagoshima, Japan; 2 Department of Cardiovascular Surgery, Graduate School of Medical and Dental Sciences, Kagoshima University, 8-35-1 Sakuragaoka, 890-8520 Kagoshima, Japan

**Keywords:** Common iliac artery aneurysm, Terminal aorta aneurysm, Saccular aneurysm, Inflammatory aneurysm, Ankylosing spondylitis, Behçet's disease, HLA-A26, HLA-B27

## Abstract

**Background:**

The human leukocyte antigen (HLA) is a major histocompatibility complex antigen found in almost all human cell membranes. Half of the HLA alleles are inherited from each parent. However, little is known about the relationship between HLA and abdominal aortic aneurysms. Herein, we present a rare surgical case of a saccular common iliac artery aneurysm associated with HLA-A26 and HLA-B27 in a young adult patient, along with a literature review.

**Case presentation:**

A 26-year-old Japanese man presented with high fever and right lower abdominal pain. Contrast-enhanced computed tomography revealed appendicitis, a 20-mm saccular aneurysm at the origin of the left common iliac artery, and an 8-mm gastric varix. After undergoing laparoscopic appendectomy, the patient was referred to our institution for management of this rare aneurysm. His mother had a history of uveitis and ankylosing spondylitis treated with steroids, suggesting that he may have inherited HLA-B27. Endovascular aortic repair was performed using an AFX2 stent graft under general anesthesia. The patient’s postoperative course was uncomplicated, and he was discharged on postoperative day three. Postoperative HLA typing revealed HLA-A11, HLA-A26, HLA-B27, and HLA-B35 positivity.

**Conclusions:**

When aortic aneurysms are incidentally found in young patients, HLA typing should be considered in addition to specific antibodies for collagen diseases. Patients with a medical or family history of collagen disease–related vasculitis and specific HLA types should undergo careful cardiovascular follow-up.

## Background

Thoracic aortic aneurysms (TAAs), abdominal aortic aneurysms (AAAs), and iliac artery aneurysms are typically caused by arteriosclerosis in elderly individuals with a history of smoking or lifestyle diseases. However, heritable thoracic aortic disease (HTAD), which includes Marfan, vascular Ehlers-Danlos, Loeys-Dietz, and Turner syndromes, occurs in young people with characteristic stature, skin, and skeletal findings, and involves well-known genes [1].

The human leukocyte antigen (HLA) is a major histocompatibility complex antigen found in almost all human cell membranes. Half of the HLA alleles are inherited from each parent. However, little is known about the relationship between HLA and AAAs [2, 3].

AAAs often occur between the renal arteries and the terminal aorta (TA), whereas common iliac artery aneurysms (CIAAs) often occur in the middle of the common iliac artery (CIA). Saccular aortic aneurysms usually occur in the descending thoracic and abdominal aortas [4]. Herein, we present a rare surgical case of saccular CIAA associated with HLA-A26 and HLA-B27 at the CIA origin in a young adult, along with a literature review.

## Case presentation

**Fig. 1:**
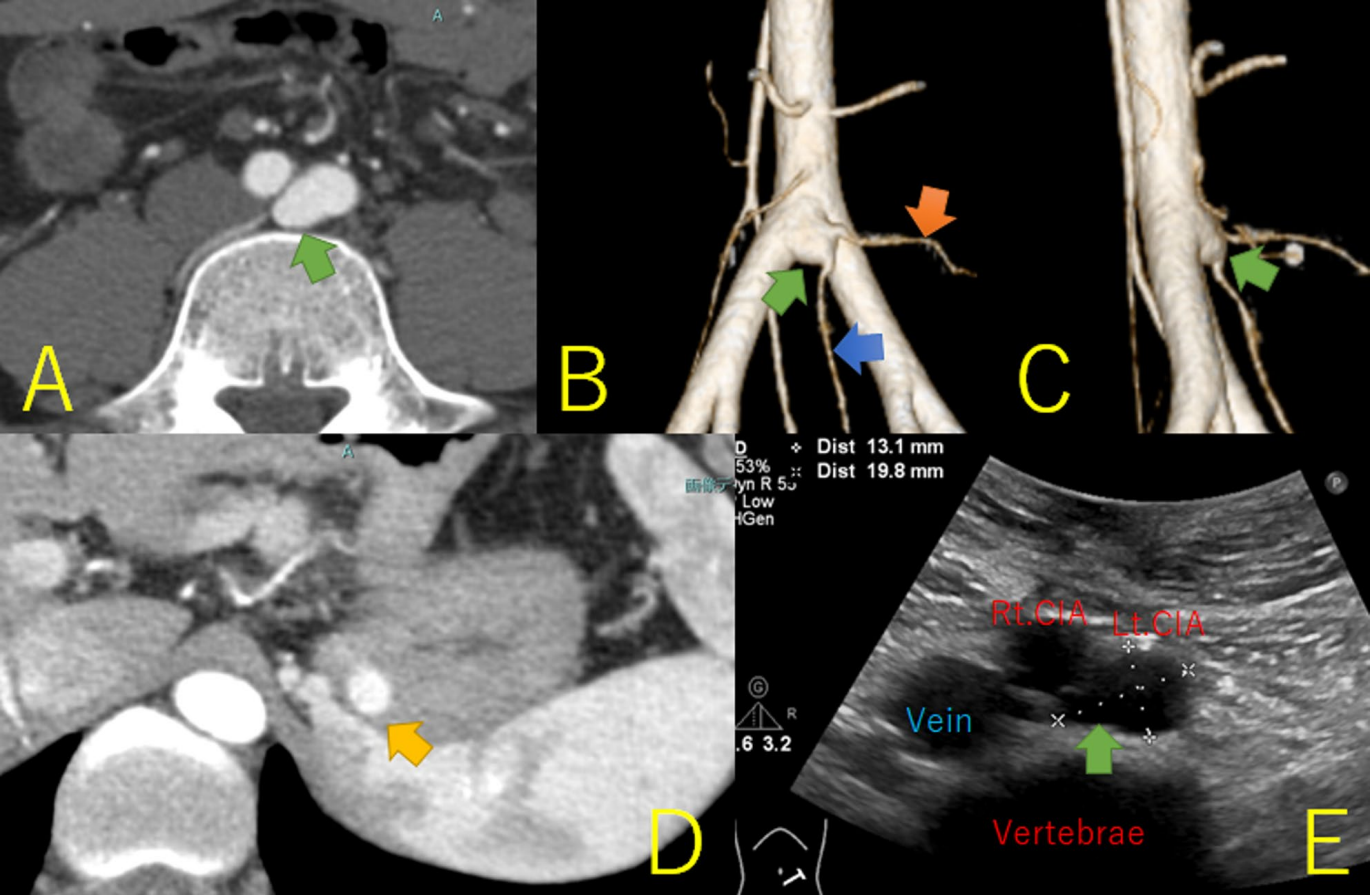
Preoperative findings. **A** Contrast-enhanced computed tomography (CT) revealing a saccular aneurysm (green arrow) measuring 20 mm in diameter at the origin of left common iliac artery(CIA) or the bifurcation of the terminal aorta. **B** Anterior-view three-dimensional CT image. The median sacral artery (blue arrow) and the left fourth lumbar artery (orange arrow) generated from the aneurysm (green arrow). **C** Left-sided three-dimensional CT image. The aneurysm (green arrow) was located at the L4/L5 level and protruded to the front of the lumbar vertebrae. **D **Gastric varix (orange arrow) measuring 8 mm in diameter **E** Ultrasonography showed the aneurysm (green arrow) measuring 13.1 × 19.8 mm in diameter at the origin of the left CIA

A 26-year-old Japanese man (height, 174 cm; weight, 81.4 kg) visited a hospital with high fever and right lower abdominal pain. He was previously healthy, without lifestyle diseases or smoking habits. Contrast-enhanced computed tomography (CT) revealed appendicitis and a 20-mm-diameter saccular aneurysm of left CIA origin or TA bifurcation (Fig. 1A and B). The aneurysm was at the L4/L5 level on the sagittal view and protruded in front of the lumbar vertebrae (Fig. 1C). No signs of mural thrombi or a mantle were observed. An 8-mm-diameter gastric varix was also observed (Fig. 1D). The abdominal aorta measured 19 mm in diameter. The median sacral and left fourth lumbar arteries originated from the aneurysm (Fig. 1B). The patient underwent an emergency laparoscopic appendectomy. Phlegmonous appendicitis with peritonitis was observed, with no findings suggestive of vasculitis or Behçet’s-related inflammation. The patient was discharged on postoperative day 4. Outpatient ultrasonography showed the aneurysm was 13.1 × 19.8 mm at the origin of the left CIA (Fig. 1E) without to-and-fro streams, suggesting that it was a true aneurysm.

The patient was referred to our institution for treatment of the rare aneurysm. A detailed medical history revealed that his mother had uveitis and ankylosing spondylitis (AS) 20 years prior to steroid treatment. She tested positive for the specific HLA (probably HLA-B27). Our patient showed no specific eye, skin, oral mucosal, or skeletal signs. Laboratory tests revealed the following: white blood cell count, 3,500/µL; C-reactive protein (CRP), 0.09 mg/dL; anti-nuclear antibody, < 40 titer (reference, < 40 titer); anti-Sjögren’s syndrome type A antibody, < 1.0 U/mL (reference, < 9.9U/mL); immunoglobulin G4, 63.9 mg/dL (reference, 11–121 mg/dL); and negativity for syphilis antibodies. Vertebral radiography revealed no specific signs of AS, such as marginal sclerosis of the sacroiliac joint, Romanus lesion, vertebral squaring, or bamboo spine.

**Fig. 2:**
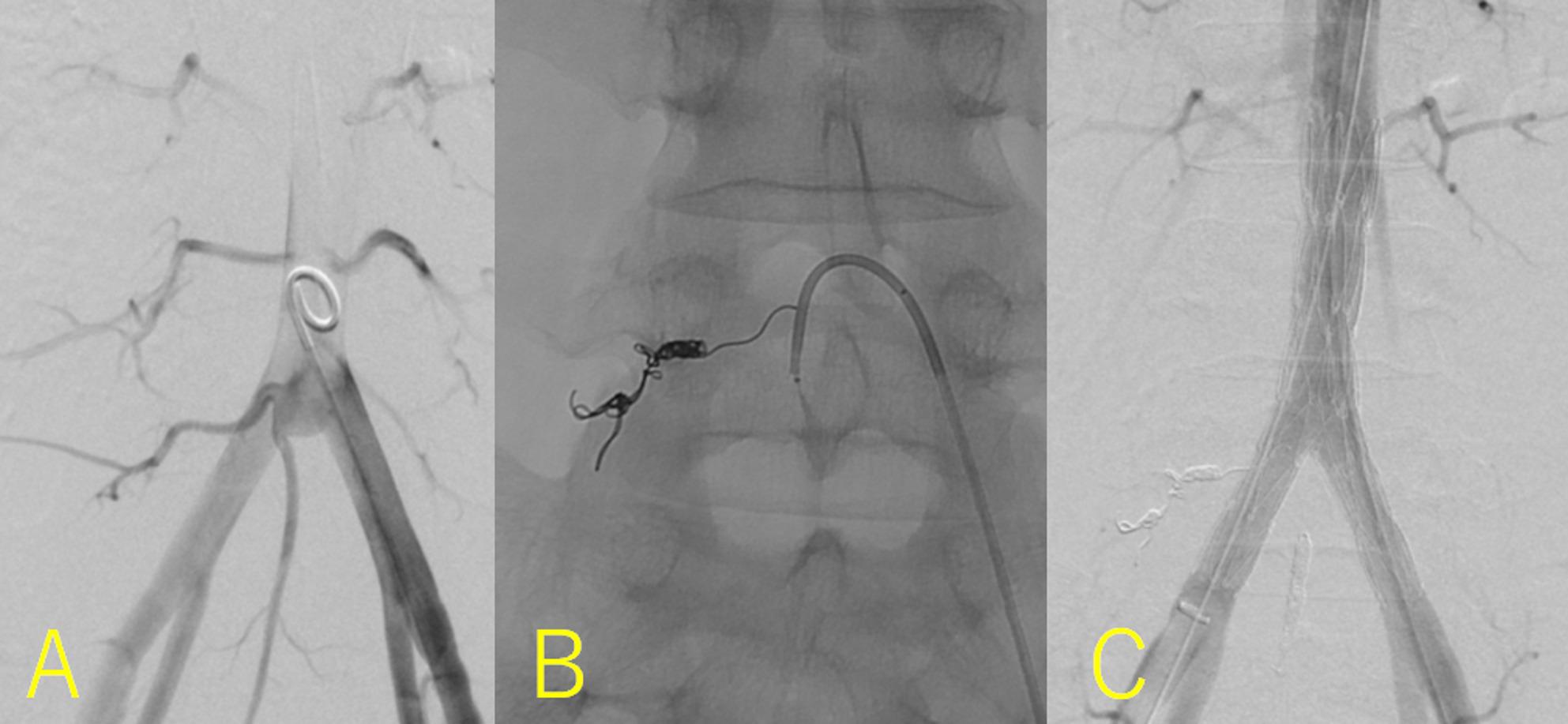
Intraoperative findings. **A** Angiography revealed the symmetrical common iliac arteries and a saccular aneurysm at the bifurcation of the terminal aorta. **B** To prevent a type II endoleak, we occluded the left fourth lumbar artery using DELTAFILLs (Johnson & Johnson, NJ, US) (5 mm × 20 cm × 2). **C** We delivered a bifurcated stentgraft AFX2 BEA 22-70/I16-30 to the terminal aorta. No endoleak was detected

**Fig. 3:**
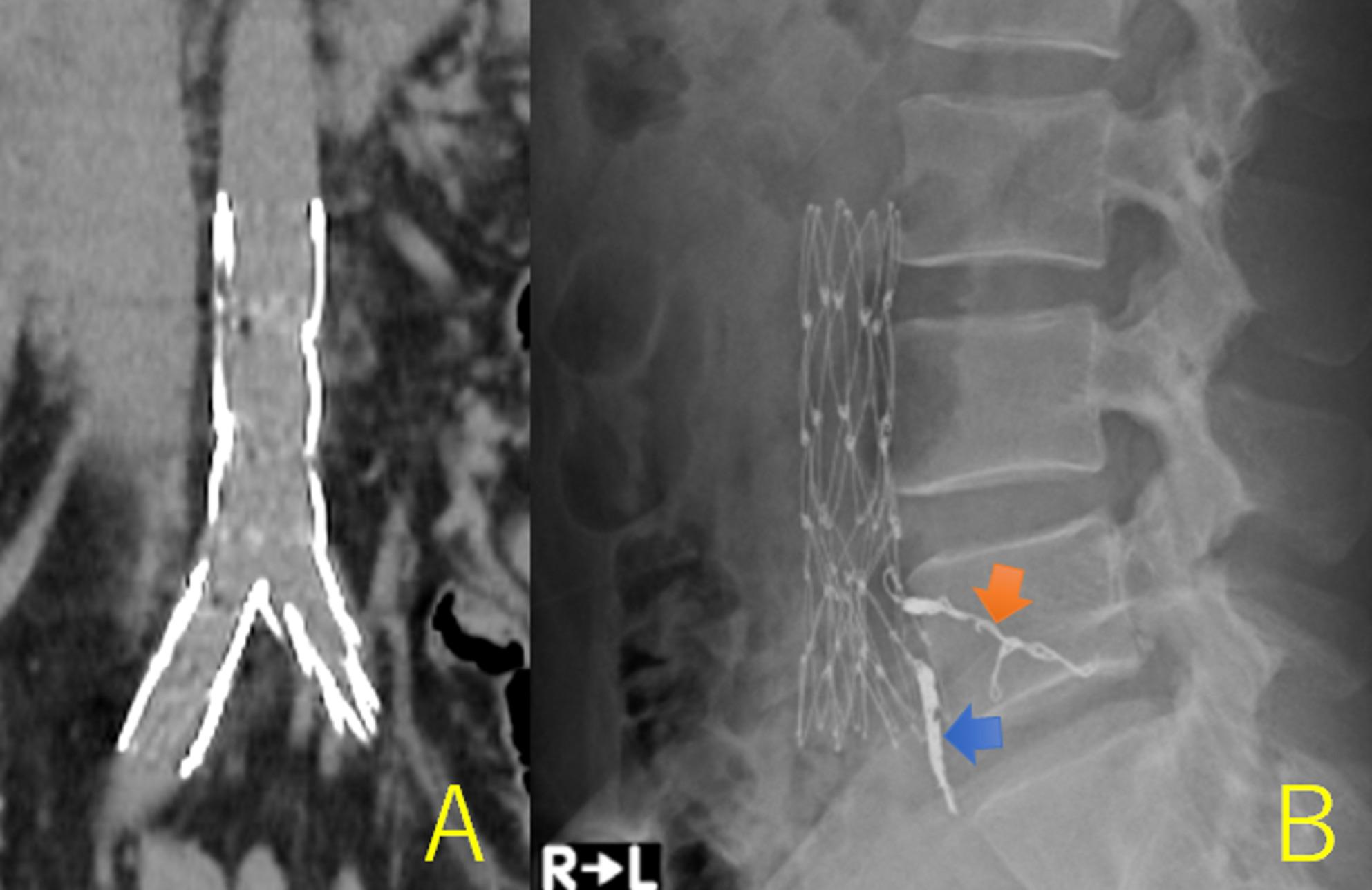
Postoperative findings.** A **Computed tomography showed no stent graft stenosis or migration. **B** A lumbar X-ray showed successful occlusion of the median sacral artery (blue arrow) and the fourth lumbar artery (orange arrow). No specific findings of ankylosing spondylitis (AS) were observed in the lumbar vertebrae or sacrum

Endovascular aortic repair (EVAR) was performed under general anesthesia. Angiography revealed symmetrical CIAs and a saccular aneurysm at the TA bifurcation (Fig. 2A). To prevent a type II endoleak, we occluded the left fourth lumbar artery using two DELTAFILLs (Johnson & Johnson, NJ, US) (5 mm × 20 cm) (Fig. 2B) and the median sacral artery using a DELTAFILL (6 mm × 25 cm) and one Nester (COOK, IN, US) (MWCE-18-14-6). Moreover, a bifurcated stent graft (AFX2 BEA 22–70/I16-30; Endologix Inc., Irvine, CA, US) was delivered to the TA. No endoleaks were observed (Fig. 2C). The operation time was 2 h and 8 min, and blood loss was 5 mL. CT and radiography revealed no stent-graft stenosis or migration (Fig. 3A and B), and the patient was discharged on postoperative day 3. Postoperative HLA typing revealed HLA-A11, HLA-A26, HLA-B27, and HLA-B35 positivity. Six months after the EVAR, CT revealed CIAA shrinkage.

## Discussion

The probability of AAAs being diagnosed in people younger than 40 years is 1.3% [5], whereas AAAs and CIAAs are rare among people in their twenties. Almost all etiologies of aortic aneurysms at a young age are not atherosclerosis but HTAD, vasculitis including vascular Behçet’s disease (BD) and Takayasu disease, and trauma [5]. Our patient had no family history of HTAD or vasculitis, and no history of abdominal trauma. If the erythrocyte sedimentation rate and pathological findings had been obtained, the incidentally detected saccular aneurysm might have been diagnosed as an inflammatory arterial aneurysm.

Half-alleles of HLA are inherited from either parent. Our patient probably inherited HLA-B27 from his mother. The prevalence of HLA-B27 in the Japanese population is 0.3–0.4% [6], and 74.8% of Japanese patients with AS have HLA-B27 [7]. AS is a chronic, progressive rheumatoid disease that primarily affects the axial skeleton. Cardiologically, AS involves aortic valve regurgitation (AR) and heart conduction disturbances including atrioventricular and bundle branch blocks. Palazzi et al. [8] reported that among patients with AS, aortitis often involves the aortic root and ascending aorta and causes aortic annulus dilation and inflammatory degeneration of the aortic cusps, leading to AR. They also postulated that extension of the subaortic fibrotic process into the interventricular septum caused conduction disturbances. Our patient did not present with AS, AR, or heart conduction disturbances, suggesting that the left CIAA may have developed independently of HLA-B27.

In contrast, HLA-A26 is associated with BD [9]. The prevalence of HLA-A26 in the Japanese population is 10.9% [10]; among Japanese patients with BD, 48.4–60% have HLA-B51, and 29.2–30% have HLA-A26 [9, 11]. BD is a chronic, relapsing disease that primarily affects the oral mucosa, skin, and eyes, and sometimes involves major organs, including the vascular, gastrointestinal, and central nervous systems. Vascular BD involves arterial and venous inflammation, which can lead to aneurysm formation and vascular occlusion. Therefore, our patient, who had a saccular CIAA and a gastric varix, may develop vascular BD in the future, which can manifest with eye, skin, oral mucosal, and joint lesions.

A search of the Ichushi-Web of Japan Medical Abstract Society, PubMed, and Google Scholar databases revealed seven reports describing eight cases of HLA-B27–positive aortic aneurysms, including TAAs and AAAs (Table 1), and two reports of HLA-A26–positive peripheral arterial aneurysms (Table 2). No reports were found of HLA–B27 positive peripheral arterial aneurysm. No reports were also found of HLA–A26 positive aortic aneurysm. In HLA-B27–positive aortic aneurysms, the average age at discovery was 45 years, and three times more men than women were affected. In all cases, AS preceded the aortic dilation and aneurysm diagnosis. All patients were symptomatic due to aortitis and AR. Aortic aneurysms develop from the sinus of Valsalva to the abdominal aorta. CRP levels are not always proportional to aneurysm size. In contrast, for HLA–A26-positive arterial aneurysms, the average age at discovery, including ours, was 34 years, and all three patients were men. The two cases involved pain at the large aneurysm site, and the aneurysms were pseudoaneurysms. The one case [20] was diagnosed with vascular BD. In vascular BDs, some aneurysms are pseudoaneurysms and multiple lesions [21]. Based on our epidemiological review of the literature, our case of iliac artery aneurysm, a type of peripheral arterial aneurysm, may be more strongly associated with HLA-A26 than with HLA-B27.

**Table 1:** Reports of HLA-B27–positive aortic aneurysms in the literature. AAA, abdominal aortic aneurysm; AR, aortic valve regurgitation; AS, ankylosing spondylitis; Asc Ao, ascending aorta; AVB, atrioventricular block; AVR, aortic valve replacement; CIAA, common iliac artery aneurysm; CRP, C-reactive protein; DAA, descending aortic aneurysm; DOE, dyspnea of exertion; ECG, electrocardiography; EVAR, endovascular aortic repair; IRBBB, incomplete right bundle branch block; MR, mitral valve regurgitation; TEVAR, thoracic endovascular aortic repair; UC, ulcerative colitis

Author	Published Year	Age/Sex	Complicated collagen diseases	Symptoms	Aneurysm type	Aneurysm diameter(mm)	Pre-operative CRP (mg/dL)	ECG	Echo-cardiography	Cardiac Angiogram	Surgery	Aortic pathology	Post-operative therapy
Kawasuji [12]	1982	46M	AS	DOE	Asc Ao dilation	Unknown	Unknown	1°AVB	Unknown	AR	AVR	Inflammation	Unknown
		53M	AS	DOE	Asc Ao aneurysm	Unknown	Unknown	1°AVB	Unknown	AR	AVR and Replacement of Asc Ao	Scarring and fragmentation	Unknown
Aoyagi [13]	1998	26M	Takayasu arteritis AS, UC	DOE	Fusiform Asc Ao aneurysm	80	18.1	Unknown	Severe AR and mild MR	Unknown	Aortic root replacement	Inflammation	Steroid
Stamp [14]	2000	29F	AS	Chest and back pain	Fusiform Asc Ao aneurysm	70	Persisting elavation	Unknown	Post AVR with homograft due to severe AR	Unknown	Replacement of the aortic valve homograft and Asc Ao with a valved aortic homograft conduit	Inflammation	Unknown
Takagi [15]	2003	68F	AS	Lower abdominal and back pain	Fusiform AAA	48	3.32	Unknown	Unknown	Unkown	Tube grafting	Inflammation	NSAIDs
Huffer [16]	2006	58M	AS	DOE	Aneurysms of the sinuses of Valsalva	55	Unknown	Unknown	Mild to moderate AR	AR1+	Aortic root replacement	Inflammation	NSAIDs
Takahashi [17]	2011	46M	AS	DOE	Fusiform Asc Ao aneurysm	50	0.15	IRBBB	severe AR	ARⅢ	Aortic root replacement	Inflammation	Unknown
Miller [18]	2017	34M	AS	Crampy abdominal pain	Fusiform ① AAA ② DAA	① 62 ② 45	57.9	Unknown	Unknown	Unknown	EVAR and TEVAR	Unavailable	Steroid

**Table 2:** Reports of HLA-A26–positive arterial aneurysms in the literature. DFA, deep femoral artery; EVT, endovascular treatment; ASA, acetylsalicylic acidCIAA, common iliac artery aneurysm

Author	Published Year	Age Sex	Complicated collagen diseases	Symptoms	Aneurysm type	Aneurysm diameter(mm)	Pre-operative CRP (mg/dL)	ECG	Echo-cardiography	Cardiac Angiogram	Surgery	Arterial pathology	Diagnosis	Post-operative therapy
Sato [19]	2015	34M	None	Thigh pain	① DFA aneurysm ② Popliteal artery aneurysm ③ Carotid artery aneurysm	①60×70 ②15×17 ③10	7.3	Unknown	Unknown	Unknown	Radical DFA ligation	Pseudo-aneurysm	Pre-vasculo-Behçet Status	Steroid Coumarin Colchicine
Abe [20]	2021	42M	Suspected RA	Clavicle pain and pulse	Impending rupture of a left subclavian artery pseudoaneurysm	65×45	15	Normal	Normal	Unknown	EVT using covered stent	Unavailable	Behçet Disease	Heparin ASA Clopidogrel Cilostazol
Our case	2025	26M	None	None	① Saccular CIAA ② Gastric varix	① 13×20 ② 8	0.09	Normal	No AR	None	EVAR	Unavailable	Unknown	None

Treatments for HLA-associated inflammatory aneurysms are diverse. Surgeries to treat HLA-B27–positive aortic aneurysms included aortic valve and root replacement, grafting, and stent grafting (Table 1), whereas those for HLA-A26–positive arterial aneurysms included direct ligation and endovascular treatment using a covered stent (Table 2). In stent grafting, including thoracic EVAR and EVAR, aortic pathology is indeterminate; however, preoperative positron emission tomography (PET) can be used to diagnose aortic inflammation [8]. In our case, PET was not performed owing to its cost. Postoperative anti-inflammatory therapies include steroids, nonsteroidal anti-inflammatory drugs, coumarins, and colchicine. In our case, the patient had no symptoms or specific laboratory findings; therefore, we decided to follow him without postoperative anti-inflammatory medications.

Patients with fusiform CIAAs > 35 mm in diameter are considered surgical candidates. In contrast, saccular CIAAs are surgical candidates regardless of their size or age [22]. Surgery for CIAA includes grafting and EVAR. In cases of inflammatory AAAs, EVAR should be considered over open surgery if the anatomy is suitable (Class IIa, evidence level C) [23]. In fact, some papers [21, 24, 25] report pseudoaneurysm formation after graft anastomosis in vasculitis-related aneurysms. Moreover, our patient hoped to return to work soon after surgery and was worried about sexual dysfunction caused by the pelvic splanchnic nerve damage. Therefore, we performed a minimally invasive EVAR procedure without complications. In terms of stent grafting, the staff at our institution usually use an Excluder (Gore, Newark, NJ, US) or AFX2 as the main body. The abdominal aorta just above the TA was narrow (15 mm in diameter), and Excluder’s legs were considered to be compressed above the TA. The AFX2, a bifurcated unibody stent graft with an endoskeletal structure, is preferred in cases of narrow TA. Therefore, we decided to use AFX2 as the primary device. The AFX2 BEA 22–70/I16-30 is the narrowest among the series [26]. The stent graft can billow and adapt to the patient’s anatomy owing to the blood pressure gradient within and around it, which is called ‘‘Active Seal.’’.

In our case, the CIAA was incidentally detected in the course of appendicitis. However, detecting aortic aneurysms before rupture is usually difficult in otherwise healthy young adults. In addition to AS, other collagen diseases, including Takayasu disease [27], BD [28], and Sjögren’s syndrome [29, 30], reportedly involve aortic aneurysms. Therefore, patients with a medical or family history of such diseases should undergo careful medical examinations using vascular ultrasonography and CT.

We treated a young adult patient with a saccular CIAA before rupture. When aortic aneurysms are found by chance in young adults, HLA typing should be considered, in addition to testing for specific antibodies against collagen diseases.

## Data Availability

Not applicable.

## Abbreviations

TAAs: Thoracic aortic aneurysms
AAAs: Abdominal aortic aneurysms
HTAD: Heritable thoracic aortic disease
HLA: Human leukocyte antigen
TA: Terminal aorta
CIAAs: Common iliac artery aneurysms
CIA: Common iliac artery
CT: Computed tomography
AS: Ankylosing spondylitis
CRP: C-reactive protein
EVAR: Endovascular aortic repair
IAAs: Iliac artery aneurysms
EVAR: Endovascular aortic repair
BD: Behçet's disease
AR: Aortic valve regurgitation
PET: Positron emission tomography
